# Prevalence of Urinary Tract Infection and Urosepsis Following Transrectal Ultrasound-Guided Prostate Biopsy: Spectrum of Pathogens and Patterns of Antibiotic Resistance

**DOI:** 10.7759/cureus.92218

**Published:** 2025-09-13

**Authors:** Muhammad Umair Zafar, Mushtaq Hussain, Asad Shahzad Hasan

**Affiliations:** 1 Urology, Nishtar Medical University, Multan, PAK; 2 Urology, Sindh Institute of Urology and Transplantation, Karachi, PAK; 3 Urology, Queen Elizabeth Hospital Birmingham, Birmingham, GBR

**Keywords:** antibiotic resistance, ciprofloxacin resistance, escherichia coli, fosfomycin sensitivity, prostate biopsy, transrectal ultrasound, urinary tract infection, urosepsis

## Abstract

Introduction

Transrectal ultrasound-guided prostate biopsy (TRUS-Bx) remains the global standard for diagnosing prostate cancer. Despite being a relatively safe procedure, it carries a risk of post-biopsy complications, most notably urinary tract infections (UTIs) and urosepsis. Increasing rates of antibiotic resistance, especially to fluoroquinolones, have raised concerns about the safety of TRUS-Bx in many regions. Data from Pakistan remain limited, prompting this study to investigate infectious complications and resistance profiles in a local tertiary care setting.

Objectives

This study aimed to determine the prevalence of UTIs and urosepsis following TRUS-Bx and to assess the spectrum of pathogens and antibiotic resistance patterns among affected patients.

Methods

This prospective descriptive cross-sectional study was conducted in the Department of Urology, Sindh Institute of Urology and Transplantation (SIUT), Karachi, from November 3, 2021, to May 2, 2022. A total of 149 male patients aged 45-80 years with lower urinary tract symptoms, elevated prostate-specific antigen (PSA > 4 ng/mL), or palpable nodules on digital rectal examination were enrolled. Patients with uncontrolled diabetes mellitus, catheterization, bladder stones, pre-biopsy UTI, or positive urine culture were excluded. Pre-biopsy rectal swabs were obtained to guide targeted antimicrobial prophylaxis. Follow-up was performed on the seventh post-procedure day with complete blood count, urine culture, and blood culture irrespective of symptoms. Data were analyzed using IBM SPSS Statistics for Windows, Version 20.0 (Released 2011; IBM Corp., Armonk, NY, USA).

Results

Among 149 patients, four (2.68%) developed UTIs, while none developed urosepsis. Urine cultures revealed bacterial growth in 69 patients (46.3%), with *Escherichia coli* being the most frequent pathogen (24.2%). Sensitivity testing showed the highest susceptibility to fosfomycin (89.9%) and the lowest to ciprofloxacin (36.2%). Blood cultures were positive in 13 patients (8.7%), most commonly with coagulase-negative *Staphylococcus* (69.2%). Overall, infectious complication rates were lower than those reported in comparable international series, but resistance to ciprofloxacin was notable.

Conclusion

The prevalence of UTI and urosepsis following TRUS-Bx in this cohort was low. However, nearly half of the patients demonstrated bacterial colonization on urine culture, with *E. coli* as the leading pathogen and ciprofloxacin resistance as a significant concern. Fosfomycin demonstrated promising sensitivity and may be considered in prophylactic regimens. These findings highlight the need for ongoing surveillance of antimicrobial resistance and optimization of biopsy prophylaxis protocols in the local population.

## Introduction

Prostate cancer is the second most common malignancy in men worldwide and a leading cause of cancer-related mortality [1]. Early and accurate diagnosis is essential, and transrectal ultrasound-guided prostate biopsy (TRUS-Bx) remains the gold standard for histological confirmation [2]. Each year, more than one million TRUS-Bx procedures are performed in the United States and Europe, with similarly high use across Asia [3]. Although generally considered safe, TRUS-Bx is associated with both minor and major complications. Hematuria and hematospermia are the most frequent minor issues, while urinary tract infection (UTI) and urosepsis represent the most clinically significant complications [4,5].

Urosepsis, though relatively uncommon, carries a disproportionately high risk of morbidity and mortality. Post-biopsy sepsis occurs in 1%-3% of cases, with severe cases linked to mortality rates approaching 30% [6]. These complications also increase healthcare costs through longer hospital stays, intensive care admissions, and use of broad-spectrum antibiotics [7].

The mechanism of infection following TRUS-Bx is primarily the inoculation of rectal flora into the prostate and urinary tract during needle passage [8]. *Escherichia coli* is the most common pathogen, followed by *Klebsiella pneumoniae* and other Enterobacteriaceae [9]. While fluoroquinolones such as ciprofloxacin have historically been used for prophylaxis [10], rising resistance has limited their effectiveness, with multidrug-resistant *E. coli* strains reported worldwide [11-13].

Recent studies show ciprofloxacin resistance in 40%-60% of *E. coli* isolates in South Asia [14], and up to 90% in parts of the Middle East, often linked to higher post-biopsy infection rates [15]. Similar trends in North America and Europe have led to increased hospitalization and morbidity [16,17]. These patterns have prompted evaluation of alternative prophylactic approaches, including pre-biopsy rectal swab-guided targeted prophylaxis and the use of fosfomycin or aminoglycosides [18,19].

Despite widespread use of TRUS-Bx in Pakistan, there is limited published data on the prevalence of post-biopsy infectious complications and local resistance patterns. A few small studies have highlighted rising rates of fluoroquinolone resistance among uropathogens, but comprehensive evaluations in the context of prostate biopsy are scarce [20,21]. Given the heavy burden of antimicrobial resistance in South Asia, understanding regional pathogen trends is critical for guiding empiric prophylaxis and minimizing complications.

This study was designed to determine the prevalence of UTI and urosepsis following TRUS-Bx in a tertiary care center in Pakistan. It also aimed to characterize the pathogens isolated from urine and blood cultures and describe their antibiotic resistance profiles, providing locally relevant evidence to support antibiotic stewardship in urological practice.

## Materials and methods

This prospective descriptive cross-sectional study was conducted in the Department of Urology, Sindh Institute of Urology and Transplantation (SIUT), Karachi, Pakistan, between November 3, 2021, and May 2, 2022. A total of 149 patients were recruited using non-probability consecutive sampling. The sample size was calculated based on a previously reported 5% prevalence of post-biopsy urosepsis [5], with a 3.5% margin of error and a 95% confidence interval. Eligible participants were men aged 45-80 years who presented with lower urinary tract symptoms with prostate-specific antigen (PSA) levels above 4 ng/mL (threshold used per institutional protocol, though age-specific reference ranges are recognized), or a palpable prostatic nodule on digital rectal examination. Patients were excluded if they had uncontrolled diabetes mellitus, indwelling catheters, bladder stones, a positive pre-biopsy urine culture, active genitourinary infection, or frank hematuria requiring hospitalization.

All patients underwent urine culture prior to biopsy, and only those with sterile results proceeded. Rectal swabs were obtained 10 days before the procedure, and antimicrobial prophylaxis was prescribed in consultation with infectious disease specialists, guided by swab sensitivity and pharmacokinetics. Commonly used agents included fluoroquinolones, fosfomycin, and aminoglycosides. TRUS-Bx was performed under local anesthesia by experienced radiologists using a 10- or 12-gauge biopsy needle, with a systematic 12-core protocol and additional cores taken when clinically indicated.

Follow-up was conducted on the seventh post-procedure day, in line with institutional protocol, as most biopsy-related infections are expected within this period. At follow-up, all patients underwent complete blood count, urinalysis, urine culture, and blood culture irrespective of symptoms. Any unscheduled visits to outpatient or emergency services were recorded for patients presenting with fever, urinary complaints, or features of sepsis. In addition, a one-month review was scheduled for all patients to detect late infectious complications; however, cultures and further investigations at this stage were only performed if patients were symptomatic. The primary outcomes were the incidence of UTI and urosepsis. UTI was defined as fever with urinary symptoms such as frequency, urgency, dysuria, suprapubic or flank pain, in combination with ≥10⁴ CFU/mL bacteriuria. Urosepsis was defined as life-threatening organ dysfunction with a positive urine or blood culture and the presence of at least two quick Sequential Organ Failure Assessment (qSOFA) criteria: respiratory rate ≥ 22/min, altered mental status, or systolic blood pressure ≤ 100 mmHg.

Antibiotic susceptibility testing of isolates was performed and interpreted according to the Clinical and Laboratory Standards Institute (CLSI) guidelines. Ethical approval was obtained from the institutional ethics committee, and written informed consent was secured from all participants. Data were analyzed using IBM SPSS Statistics for Windows, Version 20.0 (Released 2011; IBM Corp., Armonk, NY, USA). Continuous variables, including age, PSA, prostate size, and body mass index, were expressed as mean ± standard deviation or median with interquartile range. Categorical variables such as infectious complications, pathogen distribution, and resistance profiles were summarized as frequencies and percentages. The chi-square or Fisher’s exact test was used for categorical comparisons, while the independent t-test or Mann-Whitney U test was applied for continuous variables. A p-value < 0.05 was considered statistically significant.

## Results

A total of 149 men were included. The mean age was 63.8 ± 7.2 years (range: 45-80). The mean PSA was 15.6 ± 9.8 ng/mL, and the mean prostate volume was 56.4 ± 18.3 mL. Most patients had a body mass index in the overweight range (mean BMI: 26.7 ± 3.4). Baseline characteristics are shown in Table 1.

**Table 1:** Baseline demographic and clinical characteristics of patients undergoing TRUS-Bx (n = 149) Only descriptive statistics are applied. PSA: prostate-specific antigen.

Variable	Value
Age (years)	63.8 ± 7.2
PSA (ng/mL)	15.6 ± 9.8
Prostate volume (mL)	56.4 ± 18.3
BMI (kg/m²)	26.7 ± 3.4

Post-biopsy infectious complications were infrequent. Four patients (2.7%) developed symptomatic UTI as per predefined criteria, while no cases of urosepsis were observed. Urine cultures were positive in 69 patients (46.3%), though most represented asymptomatic bacteriuria. Blood cultures yielded growth in 13 patients (8.7%). Frequencies and statistical comparisons are presented in Table 2.

**Table 2:** Frequency of infectious complications after TRUS-Bx Infectious complications and culture positivity rates following TRUS-Bx. P-values shown for comparisons against negative culture groups.

Complication	Frequency (n = 149)	Percentage (%)	Statistical test	χ²/Fisher’s	p-value
Urinary tract infection	4	2.7	Chi-square	0.82	0.364
Urosepsis	0	0	–	–	–
Positive urine culture	69	46.3	Chi-square	3.14	0.076
Positive blood culture	13	8.7	Chi-square	1.22	0.269

Among the pathogens identified from urine cultures, *E. coli* was the leading organism, isolated in 24.2% of all patients, followed by *K. pneumoniae* (10.7%) and *Enterococcus faecalis* (7.4%). Other organisms, including *Proteus *and *Pseudomonas *species, were less frequent (Table 3). *E. coli* was also the most common organism isolated in patients with symptomatic UTI.

**Table 3:** Distribution of pathogens isolated from urine cultures (n = 69) Percentages are calculated from the total cohort (n = 149). *Statistically significant at p < 0.05.

Pathogen	Frequency	Percentage (%)	Statistical test	χ²	p-value
Escherichia coli	36	24.2	Chi-square	5.21	0.023*
Klebsiella pneumoniae	16	10.7	Chi-square	1.47	0.224
Enterococcus faecalis	11	7.4	Chi-square	0.96	0.327
Other organisms	6	4	Chi-square	0.44	0.508

Blood cultures were positive in 13 patients (8.7%). Coagulase-negative staphylococci (CoNS) predominated (69.2%). These were likely skin contaminants rather than true bloodstream infections, given the absence of systemic inflammatory response syndrome (SIRS) or sepsis features. No cases required intensive care admission.

Antibiotic susceptibility testing revealed marked resistance to fluoroquinolones, with only 36.2% of isolates sensitive to ciprofloxacin. In contrast, fosfomycin demonstrated excellent activity, with sensitivity in 89.9% of isolates, followed by amikacin (84.1%) and meropenem (79.7%) (Table 4). Statistical analysis confirmed that differences in sensitivity rates between fosfomycin and ciprofloxacin were highly significant (p < 0.001).

**Table 4:** Antibiotic sensitivity of urine culture isolates (n = 69) Fosfomycin and amikacin showed the highest sensitivity, while ciprofloxacin demonstrated significant resistance. *Statistically significant at p < 0.05.

Antibiotic	Sensitive n (%)	Resistant, n (%)	Statistical test	χ²	p-value
Fosfomycin	62 (89.9)	7 (10.1)	Chi-square	18.64	<0.001*
Amikacin	58 (84.1)	11 (15.9)	Chi-square	14.32	<0.001*
Meropenem	55 (79.7)	14 (20.3)	Chi-square	9.85	0.002*
Ciprofloxacin	25 (36.2)	44 (63.8)	Chi-square	7.29	0.007*

The overall analysis suggests that while post-TRUS-Bx infections were rare, bacterial colonization and antimicrobial resistance remain major concerns. The high prevalence of resistant *E. coli* highlights the limited effectiveness of fluoroquinolones for prophylaxis in this population. Fosfomycin, aminoglycosides, and carbapenems demonstrated superior efficacy and may represent better prophylactic options in similar settings.

## Discussion

This prospective study evaluated infectious complications and antibiotic resistance patterns following TRUS-Bx in a large tertiary care center in Pakistan. The overall rate of UTI was 2.7%, and no cases of urosepsis were identified. Nearly half of the patients (46.3%) demonstrated bacterial colonization on urine culture, with *E. coli* being the most common isolate. Antibiotic sensitivity patterns revealed the highest efficacy for fosfomycin, while ciprofloxacin demonstrated marked resistance.

The low incidence of clinically significant UTI and absence of urosepsis in this study contrast with international series, where post-biopsy infection rates have been reported between 2% and 6% for UTI and 1% and 3% for sepsis [1-3]. For example, a large Canadian cohort observed sepsis in 1.9% of patients following TRUS-Bx [4], while studies from Europe reported hospitalization rates of up to 4% due to post-procedural infections [5]. Our relatively low infection rate may reflect strict inclusion criteria, pre-biopsy urine culture screening, and individualized prophylaxis guided by rectal swab cultures.

Despite the low clinical complication rate, the high rate of bacterial growth on urine cultures (46.3%) is notable. Similar findings have been described in other South Asian cohorts, suggesting that bacterial colonization is common even in the absence of overt infection [6]. The clinical significance of asymptomatic bacteriuria following TRUS-Bx remains debated; however, it highlights the potential reservoir of resistant organisms in this patient population.

The predominance of *E. coli* as the leading pathogen aligns with prior reports [7-9]. Fluoroquinolone resistance in *E. coli* has emerged as a global problem, with resistance rates exceeding 40% in Asia and up to 90% in parts of the Middle East [10,11]. In our study, only 36.2% of isolates were sensitive to ciprofloxacin, confirming the limited role of fluoroquinolones in empiric prophylaxis in Pakistan. By contrast, fosfomycin, amikacin, and meropenem retained high efficacy. These findings are consistent with studies advocating the use of fosfomycin as an alternative prophylactic agent due to its broad spectrum, favorable pharmacokinetics, and minimal resistance rates [12-14].

One important consideration is the increasing interest in targeted antimicrobial prophylaxis. Several randomized trials have demonstrated that tailoring prophylaxis based on rectal swab culture significantly reduces infectious complications compared to empirical regimens [15,16]. Our use of rectal swabs prior to biopsy may have contributed to the favorable outcomes observed. Broader implementation of such strategies in Pakistan could optimize antibiotic use and reduce the risk of resistance escalation.

In addition to the standard seven-day follow-up, our cohort also underwent a one-month review to identify late infectious complications. Only patients who were symptomatic at that stage were further investigated, and no additional culture-proven UTIs or sepsis were detected. This strengthens the reliability of our findings, confirming that clinically significant infections were confined to the early post-biopsy period.

Alternative biopsy approaches are also under active investigation. Transperineal prostate biopsy has been shown to carry a substantially lower risk of infectious complications compared with the transrectal route [17,18]. Recent meta-analyses have suggested that infection-related hospitalization rates may be reduced to less than 1% with transperineal biopsy, raising the possibility of it replacing TRUS-Bx as the standard of care in high-resistance regions [19]. While resource constraints and training requirements remain barriers, these findings underscore the need to evaluate transperineal biopsy as a safer option in our local setting.

The strengths of this study include its prospective design, standardized follow-up with cultures performed in all patients regardless of symptoms, and the use of targeted prophylaxis based on rectal swabs. These factors provide a reliable estimate of both clinical infection and colonization rates. However, some limitations should be acknowledged. The single-center design may limit generalizability, and the relatively modest sample size restricts subgroup analyses (e.g., stratification by age, comorbidities, or prostate size). In addition, while rectal swabs were used, molecular resistance mechanisms such as extended-spectrum beta-lactamase (ESBL) production or carbapenem resistance were not systematically evaluated. Finally, while a one-month review was performed to capture late infectious events, longer follow-up might still be useful to detect very delayed complications.

In summary, this study adds to the limited literature from Pakistan on post-TRUS-Bx infections and resistance trends. The findings suggest that while clinical infection rates are low, colonization with resistant organisms is common, and ciprofloxacin resistance is particularly concerning. Fosfomycin and aminoglycosides remain promising prophylactic options, but ongoing surveillance and stewardship are critical. In the future, greater adoption of rectal swab-guided prophylaxis or transition to the transperineal biopsy approach may help mitigate the burden of infection and antimicrobial resistance in high-risk regions.

## Conclusions

In this prospective study, infectious complications following TRUS-Bx were infrequent, with only 2.7% of patients developing UTIs and no cases of urosepsis. However, nearly half of the cohort demonstrated bacterial growth on urine culture, most commonly *E. coli*. A strikingly high resistance rate to ciprofloxacin was observed, while fosfomycin and aminoglycosides retained high efficacy. These findings underscore the importance of region-specific antibiotic stewardship and suggest that fluoroquinolones should no longer be considered reliable prophylactic agents in our setting.

Implementation of rectal swab-guided prophylaxis and consideration of the transperineal biopsy approach may further reduce infection risks and optimize outcomes. Larger multicenter studies are warranted to validate these findings and guide evidence-based urological practice in regions burdened with antimicrobial resistance.
